# Critical speed estimated by statistically appropriate fitting procedures

**DOI:** 10.1007/s00421-021-04675-8

**Published:** 2021-04-03

**Authors:** Aurélien Patoz, Romain Spicher, Nicola Pedrani, Davide Malatesta, Fabio Borrani

**Affiliations:** 1 Institute of Sport Sciences, University of Lausanne, 1015 Lausanne, Switzerland; 2 Research and Development Department, Volodalen Swiss Sport Lab, Aigle, Switzerland

**Keywords:** Running, Curve fitting, Linear model, Hyperbolic model, Exercise prescription, Intensity domains

## Abstract

**Purpose:**

Intensity domains are recommended when prescribing exercise. The distinction between heavy and severe domains is made by the critical speed (CS), therefore requiring a mathematically accurate estimation of CS. The different model variants (distance versus time, running speed versus time, time versus running speed, and distance versus running speed) are mathematically equivalent. Nevertheless, error minimization along the correct axis is important to estimate CS and the distance that can be run above CS (*d′*). We hypothesized that comparing statistically appropriate fitting procedures, which minimize the error along the axis corresponding to the properly identified dependent variable, should provide similar estimations of CS and *d*′ but that different estimations should be obtained when comparing statistically appropriate and inappropriate fitting procedure.

**Methods:**

Sixteen male runners performed a maximal incremental aerobic test and four exhaustive runs at 90, 100, 110, and 120% of their peak speed on a treadmill. Several fitting procedures (a combination of a two-parameter model variant and regression analysis: weighted least square) were used to estimate CS and *d*′.

**Results:**

Systematic biases (*P* < 0.001) were observed between each pair of fitting procedures for CS and *d*′, even when comparing two statistically appropriate fitting procedures, though negligible, thus corroborating the hypothesis.

**Conclusion:**

The differences suggest that a statistically appropriate fitting procedure should be chosen beforehand by the researcher. This is also important for coaches that need to prescribe training sessions to their athletes based on exercise intensity, and their choice should be maintained over the running seasons.

## Introduction

Exercise intensity, one of the most important criteria for obtaining the desired metabolic stimulus and inducing specific adaptations to training (MacInnis and Gibala 2017), is often prescribed based on the percentage of the maximal rate of oxygen uptake or maximal heart rate (American College of Sports Medicine 2000; Roy et al. 2018). However, there is a large variability in the characteristics of the metabolic responses and the duration of exercise at a common percentage of the maximum between individuals. For example, Fontana et al. (2015) showed that the lactate threshold as well as critical power/speed (CP/CS) can occur at different percentages of the maximum oxygen consumption between individuals. Therefore, the control of exercise intensity is not guaranteed when the prescription is based on percentages of maximum values (DiMenna and Jones 2009; Lansley et al. 2011). Instead, Iannetta et al. (2020) recommended the use of a model that considers exercise intensity domains for exercise prescription. These different intensity domains can be defined based on the oxygen uptake kinetics (Whipp and Mahler 1980), maximum lactate steady-state (Iannetta et al. 2018), ventilatory threshold (Wasserman et al. 1973), or CP/CS (Vanhatalo et al. 2007; Jones et al. 2019).

Exercising above or below such thresholds leads to considerable differences in the physiological responses (Black et al. 2017). Therefore, training across disparate specific work intensities spanning different intensity domains is important to improve athletic performance. The CP/CS concept is widely used to evaluate the threshold intensity associated with the lower extremity of the severe intensity domain (Galán-Rioja et al. 2020; Jones et al. 2019). Therefore, having an accurate estimation of CP/CS, i.e., a very good approximation of the critical intensity but not the critical intensity per se, is important. This is usually obtained using the relationship between power/speed and time to exhaustion.

This relationship has been characterized with a number of models that differ in their mathematical form and number of parameters (Monod and Scherrer 1965; Moritani et al. 1981; Whipp et al. 1982; Morton 1996, 1986; Wilkie 1980; Peronnet and Thibault 1989). The original linear model formulation was proposed by Monod and Scherrer (1965) and relates the work performed during an exhaustive bout and the actual time to exhaustion through two parameters: the highest sustainable oxidative metabolic rate and the fixed anaerobic work capacity. The first parameter, known as CP (Monod and Scherrer 1965) or threshold of fatigue (Bigland-Ritchie and Woods 1984), separates power outputs for which exercise tolerance is predictably limited (exercise > CP) from those that can be sustained for longer periods (exercise < CP). The second parameter represents the energy reserve located in the muscle that can be utilized above CP as fast or as slow as needed (i.e., the sustainable work of exercise above that metabolic rate) (Monod and Scherrer 1965). Later, some authors related power and time to exhaustion by dividing the variables of the original model by the exercise duration (Poole et al. 1986; Gaesser and Wilson 1988; Housh et al. 1989). As exercise duration is the dependent variable and power the independent variable when considering bouts of fixed power, Gaesser et al. (1990) proposed expressing this exercise duration as a function of the power, which led to the well-known hyperbolic model (Morton and Hodgson 1996). Another model variant, proposed by Morton (2006), expresses the work performed as function of power, since this work (power multiplied by time to exhaustion) is also a dependent variable. However, this model has, to our knowledge, never been used so far.

A straightforward transposition of CP to running has been studied by several researchers (Hughson et al. 1984; Housh et al. 1991, 2001; McDermott et al. 1993). By analogy to the power versus time relationship, the running speed and time variables are related through critical speed (CS; the running analogue of CP for cycle ergometry) and anaerobic running capacity (*d';* the running analogue of the anaerobic work capacity) (Hill and Ferguson 1999; Housh et al. 1991; Hughson et al. 1984; Pepper et al. 1992). The latter was more recently and accurately defined as the distance that can be run above CS (Jones and Vanhatalo 2017). It implicitly follows that the work performed during an exhaustive bout becomes the distance travelled. These different two-parameter model variants are still extensively used to assess CS and *d*′ (for review see Jones and Vanhatalo (2017) and Jones et al. (2019)).

The estimation of CS and *d*′ are usually obtained from data provided by the critical speed test procedure (Poole et al. 1988), where the number and duration of the exhaustive runs were shown to play an important role in these estimations (Bishop et al. 1998; Mattioni Maturana et al. 2018). Based on the data provided by this test, CS and *d*′ could be estimated using a regression fitting routine. In general, the least squares (LS) loss function is used to minimize the error. In that case, the dependent variable must be observed with additive error (white noise) while the independent variable does not (Morton and Hodgson 1996). As heteroscedasticity is taking place (a smaller error is most likely to occur in the measurement of time to exhaustion for high running speeds, i.e., for short times to exhaustion (McLellan and Skinner 1985; Poole et al. 1988; Faude et al. 2017)), Morton and Hodgson (1996) suggested using weighted LS (WLS) in the regression analysis with weights proportional to the inverse of the variance of time to exhaustion, where the variance is itself proportional to the time to exhaustion.

The different model variants (distance versus time, running speed versus time, time versus running speed, and distance versus speed) are mathematically equivalent. Nevertheless, error minimization along the correct axis is important to estimate CS and *d*′, as already highlighted but not yet investigated by Gaesser et al. (1995). Therefore, the purpose of this study was to compare the estimations of CS and *d*′ obtained using statistically appropriate fitting procedures (which minimize the error along the axis corresponding to the properly identified dependent variables (Vinetti et al. 2020)), and statistically inappropriate fitting procedures (which do not minimize the error along the axis that contain the dependent variable) but are frequently used in the literature (Jones et al. 2019; Jones and Vanhatalo 2017). These estimations were obtained using several combinations of a linear two-parameter model variant and a regression analysis (fitting procedure). We hypothesized that the comparison of statistically appropriate fitting procedures should provide similar estimations of CS and *d*′. On the other hand, different estimations of CS and *d*′ should be obtained when comparing a statistically appropriate with a statistically inappropriate fitting procedure.

## Materials and methods

### Participant characteristics

Sixteen male runners participated in the present experiment (age: 25.6 ± 3.9 years old; height: 1.79 ± 0.05 m; body mass: 69.2 ± 5.3 kg; speed associated with maximum oxygen consumption (${s}_{\dot{\mathrm{V}}{\mathrm{O}}_{2}\mathrm{max}}$): 18.2 ± 1.4 km/h; maximum oxygen consumption: 63.0 ± 4.9 ml/min/kg). For study inclusion, participants were required to be in good self-reported general health with no symptoms of cardiovascular disease or major coronary risk factors, no current or recent lower-extremity injury that could prevent them from giving 100% of their capacity during the test and to meet a certain level of running performance. More specifically, runners were required to have an ${s}_{\dot{\mathrm{V}}{\mathrm{O}}_{2}\mathrm{max}}$ greater or equal to 16 km/h.

### Experimental procedure

Each participant completed five experimental sessions interspersed by at least two days in the laboratory. All participants were advised to avoid strenuous exercise the day before a test but to maintain their usual training programme otherwise. During the first session, participants completed a maximal incremental aerobic test on a treadmill (Arsalis T150—FMT-MED, Louvain-la-Neuve, Belgium). This test consisted of a 10-min warm-up at 10 km/h followed by an incremental increase in the running speed of 1 km/h every two minutes until exhaustion. This test was used to determine the peak speed (PS) of the incremental test of each participant. PS is defined as the running speed of the last fully completed increment (${s}_{\dot{\mathrm{V}}{\mathrm{O}}_{2}\mathrm{max}}$) plus the fraction of time spent in the following uncompleted increment ($\alpha$) multiplied by the running speed increment (*∆s* = 1 km/h) (Kuipers et al. 2003): $\mathrm{PS}={s}_{\dot{\mathrm{V}}{\mathrm{O}}_{2}\mathrm{max}}+\alpha \Delta s.$

The other four tests were performed in a randomized order and consisted of exhaustive runs at a given percentage of the participant's PS (90, 100, 110, or 120%). These tests were as follows: after a 10-min warm-up at 10 km/h and a 5-min rest period, the running speed was increased to a given percentage of PS, and the participant had to maintain the pace until exhaustion. The time to exhaustion was collected for each of the four sessions. No information about the timings or running speed was given to any participant during any of the five experimental sessions. All participants were familiar with running on a treadmill.

### Mathematical modelling

The estimations of CS and *d*′ were obtained from the following four different but mathematically equivalent equations

1 $$t\left(s\right)=\frac{{d}^{{\prime}}}{s-\mathrm{CS}}$$

2 $$d(s)=s\frac{{d}^{{\prime}}}{\mathrm{s}-\mathrm{CS}}$$

3 $$s\left(t\right)=\frac{{d}^{{\prime}}}{t}+\mathrm{CS}$$

4 $$d(t)={d}^{{\prime}}+\mathrm{CS} t$$

where $t$, *s*, and *d* stand for time to exhaustion, running speed, and distance, respectively. Equation 4 represents the original linear model of Monod and Scherrer (1965). Whipp et al. (1982) and Gaesser et al. (1990) proposed the models given by Eqs. 3 and 1, respectively. Equation 2 denotes the distance as function of running speed model proposed by Morton (2006).

### Data analysis

Four different fitting procedures were used on the data set obtained for each subject to estimate CS and *d*′. More specifically, $t\left(s\right)$ (Eq. 1) using WLS and $d\left(s\right)$ (Eq. 2) using WLS were evaluated. These first two fitting procedures are statistically appropriate. The two other fitting procedures that have been evaluated were $s(t)$ (Eq. 3) using LS and $d(t)$ (Eq. 4) using LS. These two fitting procedures are statistically inappropriate but are frequently used in the literature (Jones et al. 2019; Jones and Vanhatalo 2017). In the first case, time to exhaustion should be considered as the dependent variable and not speed. In the second case, both distance and time to exhaustion should be considered as dependent variables and not only distance. However, the errors of both variables are correlated, i.e., the error of distance is given by the product of speed and the error of time to exhaustion variable, since speed does not carry any error. This is known as endogeneity and, to the best of our knowledge, there exists no regression method that can handle such case (Antonakis et al. 2014). Weights were applied to corresponding dependent variables (time to exhaustion or distance) only in the statistically appropriate fitting procedures. Error minimization was performed iteratively using the Levenberg–Marquardt algorithm (Levenberg 1944; Marquardt 1963) for (*W*)LS regression. The standard error of the estimate (SEE) in absolute numbers for both CS and *d*′, the combined SEE (%SEE), i.e., the sum of SEE of CS and *d*′ transformed to percent units, and the residual standard error (RSE) of the fitting procedure were computed to assess the quality of the fit. Data analysis was performed using Python (version 3.7.4, Python Software Foundation. Available at http://www.python.org).

### Statistical analysis

Descriptive statistics are presented using mean ± standard deviation (SD) unless otherwise indicated. The normality of the data was confirmed through the Shapiro–Wilk test. Bland–Altman plots were constructed to examine the presence of systematic and proportional bias on CS and *d*′ estimated from two different fitting procedures (Bland and Altman 1995; Atkinson and Nevill 1998). Systematic bias was also identified by a significant difference obtained from a paired two-sided Student’s *t*-test. After confirming no correlation amongst the residuals using the Durbin-Watson test (Durbin-Watson statistic between 1.5 and 2.5), the proportional bias (heteroscedasticity) was identified by a significant slope of the regression line. In addition, the estimations of CS and *d*′ obtained from the two statistically appropriate fitting procedures as well as from a statistically appropriate and both statistically inappropriate fitting procedures were compared using one-way repeated measures ANOVA (RM-ANOVA) with Mauchly’s correction for sphericity and employing Holm corrections for pairwise post hoc comparisons. Statistical analysis was performed using Jamovi (version 1.0.8, [Computer Software], retrieved from https://www.jamovi.org) and R (version 3.5.0, The R Foundation for Statistical Computing, Vienna, Austria) with a level of significance set at *P* ≤ 0.05.

## Results

Table 1 depicts the time to exhaustion corresponding to the four exhaustive runs performed at 90, 100, 110, and 120% of the participant's PS.

**Table 1:** Means ± standard deviations of the time to exhaustion corresponding to the four exhaustive runs performed at 90, 100, 110, and 120% of the participant's peak aerobic speed (PS)

Running speed (%PS)	90	100	110	120
Time to exhaustion (min)	14.8 ± 2.57	5.94 ± 1.21	2.78 ± 0.78	1.68 ± 0.50

Table 2 depicts the estimations of CS and *d*′ obtained from the two statistically appropriate [$t(s)$ using WLS and $d\left(s\right)$ using WLS] and the two statistically inappropriate but frequently used [$s(t)$ using LS and $d(t)$ using LS] fitting procedures together with their corresponding %SEE and RSE. Note that as the units of the residual sum of squares depend on the fitting procedure itself, the RSE cannot be compared between the different fitting procedures employed. The smallest to largest estimations of CS were given by $t(s)$ using WLS and $d(s)$ using WLS (same CS), $d(t)$ using LS, and $s(t)$ using LS, while those for *d*′ were ordered as $s(t)$ using LS, $d(t)$ using LS, $d(s)$ using WLS, and $t(s)$ using WLS (Table 2).

**Table 2:** Means ± standard deviations of the critical speed (CS) and distance that can be run above CS (*d*′) and their corresponding standard error of estimate (SEE, in parenthesis) obtained from statistically appropriate [$t(s)$ using weighted least squares (WLS) and $d(s)$ using WLS] and statistically inappropriate [$s(t)$ and $d(t)$ both using LS] fitting procedures together with the combined SEE (%SEE), i.e., the sum of SEE of CS and *d*′ transformed to percent units, as well as the residual standard errors (RSE)

Statistically appropriate	Fitting procedure	CS (m/s)	*d*′ (m)	%SEE	RSE
Yes	$t(s)$ using WLS	4.39 ± 0.41 (0.03 ± 0.01)	226.0 ± 57.0 (20.3 ± 8.0)	9.8 ± 3.4	37.0 ± 14.5
	$d(s)$ using WLS	4.39 ± 0.40 (0.03 ± 0.01)	222.3 ± 56.0 (19.8 ± 7.6)	9.7 ± 3.4	201.5 ± 79.3
No	$s(t)$ using LS	4.59 ± 0.43 (0.07 ± 0.02)	167.3 ± 46.2 (11.2 ± 4.3)	8.3 ± 2.6	0.11 ± 0.04
	$d(t)$ using LS	4.42 ± 0.39 (0.04 ± 0.02)	210.2 ± 50.5 (19.7 ± 7.7)	10.5 ± 3.9	34.4 ± 11.9

### Comparison between statistically appropriate [$t(s)$ using WLS and $d(s)$ using WLS] fitting procedures

**Fig. 1:**
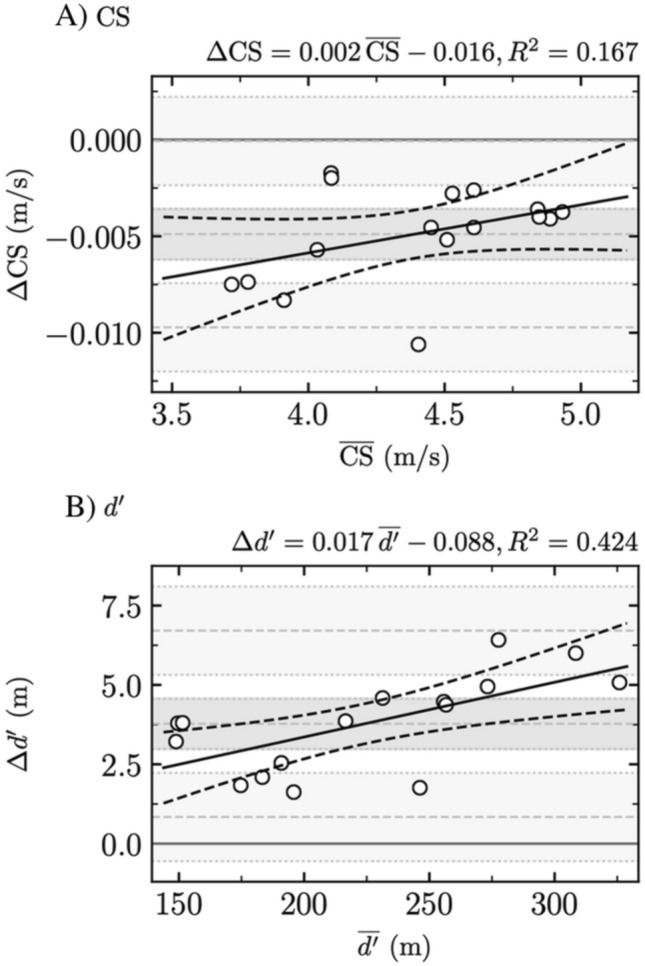
Comparison between statistically appropriate fitting procedures. Bland–Altman plots comparing $t(s)$ using weighted least squares (WLS) and $d(s)$ using WLS for (i) critical speed (CS) and (ii) distance that can be run above CS (*d*′)

Bland–Altman plots comparing statistically appropriate fitting procedures for both CS and *d*′ are depicted in Fig. 1, while Table 3 reports their systematic and proportional biases.

**Table 3:** Systematic bias ± random error (RE, i.e., 1.6 standard deviation) and proportional bias ± residual standard error (RSE) for critical speed (CS) and distance that can be run above CS (*d*′) when comparing statistically appropriate fitting procedures, i.e., $t(s)$ using weighted least squares (WLS) and $d(s)$ using WLS

	$t\left(s\right)$ using WLS vs. $d(s)$ using WLS	
	CS	*d*′
Systematic bias ± RE *P*	**− 0.005 ± 0.001** ** < 0.001**	**3.8 ± 0.8** ** < 0.001**
Proportional bias ± RSE *P*	0.002 ± 0.001 0.12	**0.02 ± 0.005** **0.006**

Significant differences (*P* ≤ 0.05) are depicted in bold font

### Comparison between the statistically appropriate [$t\left(s\right)$ using WLS] and the two statistically inappropriate [$s\left(t\right)$ and $d(t)$ both using LS] fitting procedures

**Fig. 2:**
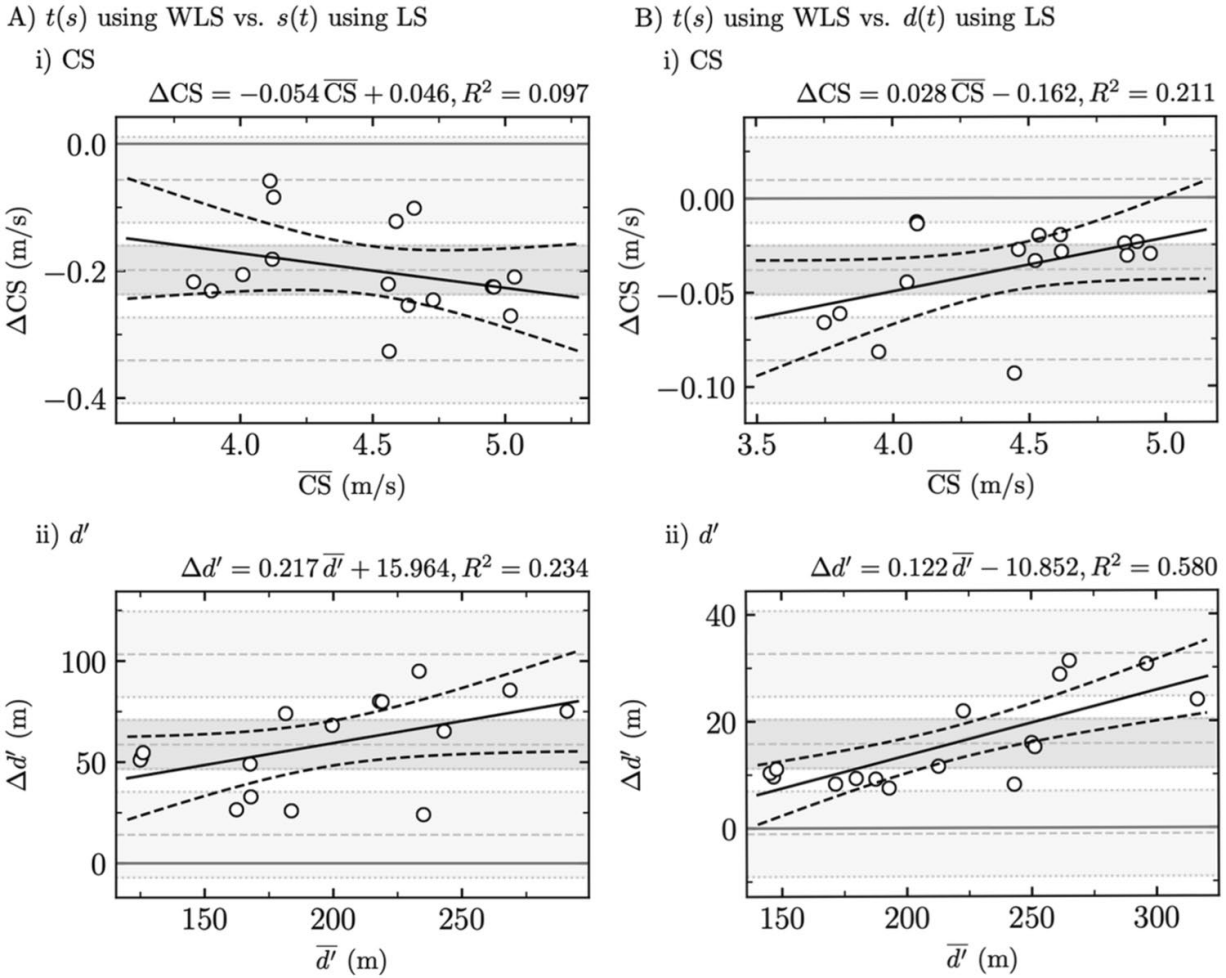
Comparison between the statistically appropriate [$t(s)$ using weighted least squares (WLS)] and the two statistically inappropriate fitting procedures. Bland–Altman plots comparing **a** $t(s)$ using WLS and $s(t)$ using LS and **b** $t(s)$ using WLS and $d(t)$ using LS for (i) critical speed (CS) and (ii) distance that can be run above CS (*d*′)

Bland–Altman plots comparing the statistically appropriate $t(s)$ using WLS fitting procedure to the two frequently used but statistically inappropriate fitting procedures for both CS and *d*′ are depicted in Fig. 2, while Table 4 reports their systematic and proportional biases.

**Table 4:** Systematic bias ± random error (RE, i.e., 1.6 standard deviation) and proportional bias ± residual standard error (RSE) for critical speed (CS) and distance that can be run above CS (*d*′) when comparing $t(s)$ using weighted least squares (WLS) with both $s(t)$ using least squares (LS) and $d(t)$ using LS

	$t(s)$ using WLS vs. $s\left(t\right)$ using LS		$t(s)$ using WLS vs. $d(t)$ using LS	
	CS	*d*′	CS	*d*′
Systematic bias ± RE *P*	**− 0.20 ± 0.04** ** < 0.001**	**58.7 ± 12.2** ** < 0.001**	**− 0.04 ± 0.01** ** < 0.001**	**15.8 ± 4.6** ** < 0.001**
Proportional bias ± RSE *P*	− 0.05 ± 0.04 0.24	0.22 ± 0.11 0.06	0.03 ± 0.01 0.07	**0.12 ± 0.03** ** < 0.001**

Significant differences (*P* ≤ 0.05) are depicted in bold font

The comparison of the three fitting procedures using RM-ANOVA yielded a significant main effect (*P* < 0.001) for both CS and *d*′. In addition, post hoc comparisons gave significant differences between each pair of fitting procedures and for both CS and *d*′ (*P* ≤ 0.01). Notably, the pair [$t(s)$ using WLS, $d(t)$ using LS] was the only comparison giving *P* values larger than 0.001 for CS, i.e., 0.01.

### Comparison between the statistically appropriate [$d(s)$ using WLS] and the two statistically inappropriate [$s(t)$ and $d(t)$ both using LS] fitting procedures

**Fig. 3:**
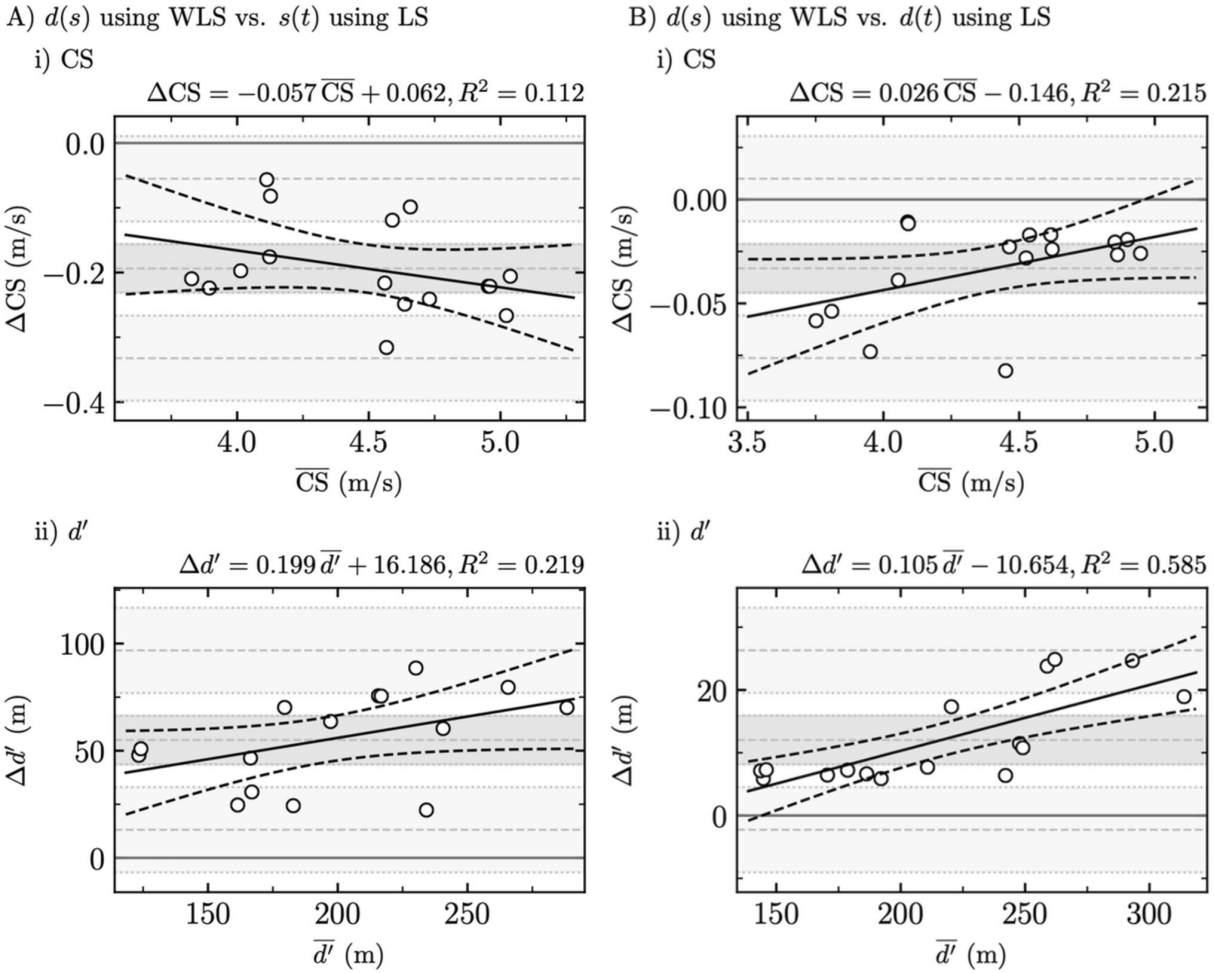
Comparison between the statistically appropriate [$d(s)$ using weighted least squares (WLS)] and the two statistically inappropriate fitting procedures. Bland–Altman plots comparing **a** $d(s)$ using WLS and $s(t)$ using LS and **b** $d(s)$ using WLS and $d(t)$ using LS for (i) critical speed (CS) and (ii) distance that can be run above CS (d')

Bland–Altman plots comparing the statistically appropriate $d(s)$ using WLS fitting procedure to the two frequently used but statistically inappropriate fitting procedures are depicted in Fig. 3, while Table 5 reports their systematic and proportional biases.

**Table 5:** Systematic bias ± random error (RE, i.e., 1.6 standard deviation) and proportional bias ± residual standard error (RSE) for critical speed (CS) and distance that can be run above CS (*d*′) when comparing $d(s)$ using weighted least squares (WLS) with both $s(t)$ using least squares (LS) and $d(t)$ using LS

	$d(s)$ using WLS vs. $s\left(t\right)$ using LS		$d(s)$ using WLS vs. $d(t)$ using LS	
	CS	*d*′	CS	*d*′
Systematic bias ± RE *P*	**− 0.19 ± 0.04** ** < 0.001**	**55.0 ± 11.3** ** < 0.001**	**− 0.03 ± 0.01** ** < 0.001**	**12.0 ± 3.9** ** < 0.001**
Proportional bias ± RSE *P*	− 0.06 ± 0.04 0.21	0.20 ± 0.10 0.07	0.03 ± 0.01 0.07	**0.11 ± 0.02** ** < 0.001**

Significant differences (*P* ≤ 0.05) are depicted in bold font

The comparison of the three fitting procedures using RM-ANOVA yielded a significant main effect (*P* < 0.001) for both CS and *d*′. In addition, post hoc comparisons yielded significant differences between each pair of fitting procedures and for both CS and *d*′ (*P* ≤ 0.02). Notably, the pair [$d(s)$ using WLS, $d(t)$ using LS] was the only comparison giving *P* values larger than 0.001 for CS and *d’*, i.e., 0.02 and 0.006, respectively.

## Discussion

Conventional statistical approaches demonstrated a systematic bias between each pair of fitting procedures for the estimation of both CS and *d*′. These results were in line with the hypothesis that different estimations of CS and *d*′ should have been obtained when comparing a statistically appropriate with a statistically inappropriate fitting procedure. Although these findings seem to refute the hypothesis that similar estimations of CS and *d*′ should have been obtained when comparing statistically appropriate fitting procedures, the differences for these estimations between statistically appropriate fitting procedures were negligible.

As pointed out by Iannetta et al. (2020), coaches are recommended to prescribe exercise based on intensity domains. To do so, one possibility is to estimate CS and use it as a limit between the heavy and severe intensity domains (Jones et al. 2019). Therefore, an accurate estimation of CS is required. There exist two statistically appropriate fitting procedures for the two-parameter model variants that allow us to estimate CS: $t(s)$ using WLS and $d(s)$ using WLS. The comparison of these two fitting procedures yielded significant systematic biases − 0.005 ± 0.001 m/s (0.018 ± 0.004 km/h) and 3.8 ± 0.8 m for CS and *d*′, respectively (*P* < 0.001). However, the bias for CS was less than treadmills’ speed resolution. Therefore, these differences could be assumed to be negligible when prescribing a training session based on exercise intensity because they would be practically meaningless. Nonetheless, they could be due to the specific data set used in this study and could potentially be larger with another data set, other choices of running speeds, a larger number of exhaustive runs, or another underlying model (e.g., three-parameters or exponential). In addition, even though the estimated CS should be a very good approximation of the critical intensity but not the critical intensity per se, we suggest coaches to physiologically verify that the estimated CS represents the upper boundary of sustainable exercise. Moreover, there is a day-to-day variation in human performance and given the SEE of CS (0.03 ± 0.01 m/s or 0.11 ± 0.04 km/h, Table 2), its confidence limits are about 10% of its value. Therefore, it would be justified to prescribe exercise intensity outside these confidence limits to avoid being in a range of values that are uncertain due to measurement error, which could be defined as the phase transition between heavy and severe intensity domains (Anderson et al. 2019). From a practical perspective, coaches could still prescribe exercise intensity at CS, but should acknowledge that there might be a source of error, especially if no physiological verification was performed.

The comparison between the statistically appropriate $t(s)$ using WLS [or $d(s)$ using WLS] and statistically inappropriate $d(t)$ using LS fitting procedures produced systematic but reasonably small biases for both CS (< − 0.04 ± 0.01 m/s; 0.14 ± 0.04 km/h) and *d*′ (< 15.8 ± 4.6 m). These differences are quite small and could be assumed to be negligible. The largest biases were obtained between the statistically appropriate $t(s)$ using WLS [or $d(s)$ using WLS] and statistically inappropriate $s(t)$ using LS fitting procedures (CS: < − 0.20 ± 0.04 m/s or 0.72 ± 0.14 km/h, *d*′: < 58.7 ± 12.2 m). In this case, the observed differences could have an impact when prescribing a training session based on exercise intensity. Nonetheless, as previously mentioned already, the magnitude of all the observed differences could be due to the specific data set and could potentially be smaller or larger. Moreover, as all comparisons of fitting procedures yielded systematic biases, it suggests that each fitting procedure produced specific estimations of CS and *d*′. Therefore, we encourage coaches to verify that the estimated CS coincide with the physiological CS and make small adjustments based on the observed performance.

The coefficient of determination is not a reliable measure to assess the goodness of fit when using WLS (Willet and Singer 1988; Kvalseth 1985). Therefore, one possibility is to use the residual sum of squares or a parameter that depends on it, such as RSE. However, the units of RSE depend on the fitting procedure and, more specifically, on the choice of the vertical and horizontal axes for the model variant and on which axes the errors are being minimized, making it impossible to compare the RSE of different fitting procedures. Moreover, when the time to exhaustion is assumed to be the independent variable, a lower RSE is necessarily observed because the data points mostly lied in the region where there was a high difference between the measured and predicted data in the horizontal axis (time to exhaustion variable) but a small difference in the vertical axis (running speed or distance variable). Therefore, a lower RSE and thus a perception of a better fitting procedure is likely to be provided by assuming the running speed or distance as the dependent variable instead of the time to exhaustion (Vinetti et al. 2020). In the case of distance as function of time, even if distance is indeed a dependent variable, error minimization only along the vertical axis (distance variable) is not statistically appropriate and there exists no regression method that can take into account the fact the errors are actually correlated. On the other hand, one could use %SEE and assume that the smallest %SEE provides the best fit quality (Triska et al. 2021). However, obtaining lower RSE or %SEE are not consistent with the experiment generating the data set but with the representation of the data set itself, as already pointed out by Vinetti et al. (2020). Therefore, based on these observations, we suggest deciding the choice of regression analysis and model variant beforehand. Moreover, this choice should be based on the specific data set (the sources of experimental error) to lead to a statistically appropriate fitting procedure. Then, we suggest to physiologically verify that the estimated CS represents a very good approximation of the actual CS.

Heteroscedasticity of the dependent variable was explicitly depicted by Hinckson and Hopkins (2005) when using usual LS fitting procedure. Indeed, these authors demonstrated systematic and nonuniform deviation from their models by showing the residuals as function of predicted values. In this study, the suggestion made by Morton and Hodgson (1996) to include weights to overcome heteroscedasticity was applied.

### Practical applications

The preferred choice between model variants is not clear (Gaesser et al. 1990; Hill 1993) and researchers/coaches might be confused on which model variant to select and the corresponding regression analysis to apply based on their data set. Therefore, a methodology to select a statistically appropriate fitting procedure is provided. The following methodology specifically addresses running speed and distance, but any occurrence of these terms can be replaced by power and work, respectively. Moreover, special cases that need to be taken into account when dealing with power or work are explicitly mentioned. Furthermore, the methodology is presented using WLS regression applied to the two-parameter model variants. This methodology can be generalized to other choices of loss functions and more complicated (e.g., three-parameter or exponential) models.

**Fig. 4:**
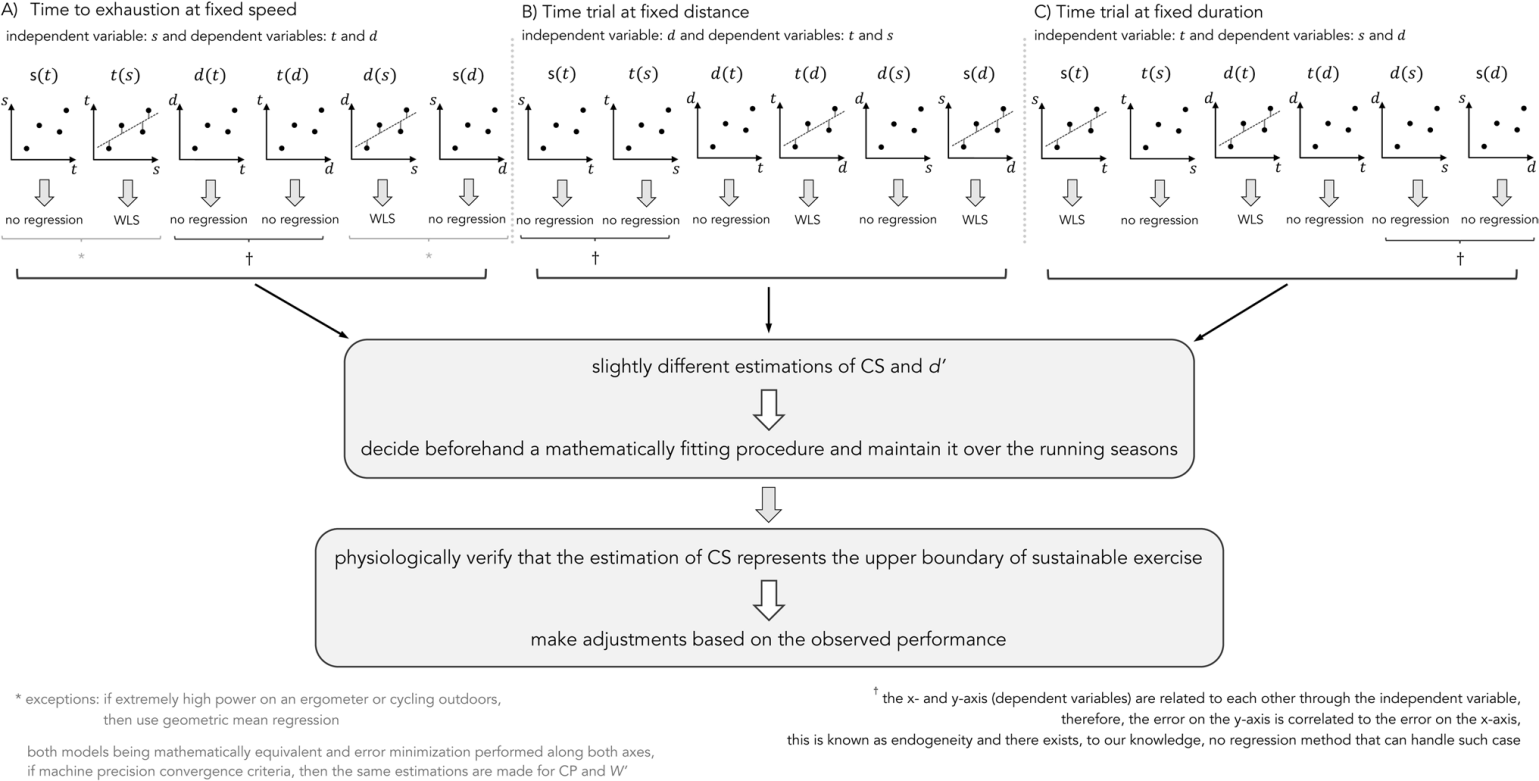
Recommendations on the choice of regression analysis. **a** Time to exhaustion (dependent variable: *t*) is measured for a fixed running speed (*s*). Distance (*d*) is by induction a dependent variable. **b** Time trial and running speed (dependent variables) are measured for a fixed distance (independent variable). **c** Distance and running speed (dependent variables) are measured for a fixed time trial (independent variable). For sake of clarity, the models represented in the figures are not representative of the outcome of the measurements. They are only given to demonstrate where a regression method can be applied. *WLS* weighted least squares, *CS* critical speed, *d*′ distance that can be run above CS, *CP* critical power, *W*′ anaerobic work capacity

First, an experiment that fixes running speed (independent variable: *s*) and measures time to exhaustion and distance (dependent variables: *t* and *d*) is considered (Fig. 4a). Special consideration exists in the case of extremely high power on an ergometer or when cycling outdoors (Vinetti et al. 2020; Maier et al. 2017). In such cases, power should be considered as a dependent variable and geometric mean regression should be employed. The recommendations on the choice of the regression analysis are as follows:

1. No regression analysis should be used with the models $s(t)$ and $s(d)$ [the inverse function of $d(s)$] because in these cases, *t* and *d*, respectively, should be the dependent variables, but they are not. In the case of extremely high power on an ergometer or when cycling outdoors, geometric mean regression should be used (Vinetti et al. 2020).
2. WLS should be used with the models $t(s)$ and $d(s)$ with weights applied to $t$ and $d$, respectively. In the case of extremely high power on an ergometer or when cycling outdoors, geometric mean regression should be used (Vinetti et al. 2020).
3. No regression should be used with the models $d(t)$ and $t(d)$ [the inverse function of $d(t)$] as the errors are correlated and no regression method exists to handle such case.

Second, an experiment that fixes distance (independent variable: *d*) and measures time trial and running speed (dependent variables: *t* and *s*) is considered (Fig. 4b). The recommendations are as follows:

1. No regression analysis should be used with the models $d(t)$ and $d(s)$ because in these cases, *t* and *s,* respectively, should be the dependent variables, but they are not.
2. WLS should be used with the models $t(d)$ and $s(d)$ with weights applied to $t$ and $s$, respectively.
3. No regression should be used with the models $s(t)$ and $t(s)$ as the errors are correlated and no regression method exists to handle such case.

Third, an experiment that fixes time (independent variable: *t*) and measures running speed and distance (dependent variables: *s* and *d*) is considered (Fig. 4c). The recommendations are as follows:

1. No regression analysis should be used with the models $t(s)$ and $t(d)$ because in these cases, *s* and *d,* respectively, should be the dependent variables, but they are not.
2. WLS should be used with the models $s(t)$ and $d(t)$ with weights applied to $s$ and $d$, respectively.
3. No regression should be used with the models $d(s)$ and $s(d)$ as the errors are correlated and no regression method exists to handle such case.

Of note, we did not consider WLS that estimates parameters based on an error minimization along the horizontal axis of a given model $f(\cdot )$. The reason being that using WLS based on an error minimization along the horizontal axis is equivalent to applying the usual WLS regression on ${f(\cdot )}^{-1}$, i.e., the inverse of the model variant. However, it should be pointed out that if ${f(\cdot )}^{-1}$ does not exist (the function is not invertible), then WLS based on an error minimization along the horizontal axis of the model variant $f(\cdot )$ should be used.

Finally, potential error in the model should be acknowledged and a physiological verification that the estimated CS represents the upper boundary of sustainable exercise should be made. In addition, small adjustments based on the observed performance could be applied.

### Methodological limitations

A few limitations to the present study are worth noting. First, no test–retest repeatability of time to exhaustion has been performed. However, even if repeatability was shown to have up to 15% error (Laursen et al. 2007), correctly assigning variables being dependent on time to exhaustion, as the dependent variables, automatically takes into account the fact that they carry error. Nonetheless, familiarization was shown to increase reliability but tends to be quite unpractical for the participant (Triska et al. 2017). Second, no runs below, at, and above CS whilst assessing oxygen uptake responses to exercise were performed to physiologically verify that the estimated CS obtained with statistically appropriate fitting procedures represents the threshold intensity associated with the lower extremity of the severe intensity domain. Although this is beyond the aim of this study, future studies, using these runs, may determine it. Third, the selected percent of PS (90, 100, 110, and 120%) resulted in time distributions that were relatively unbalanced. Therefore, the estimated CS might not represent the physiological CS (Bishop et al. 1998; Mattioni Maturana et al. 2018). However, this did not affect the present study as the main goal was not to show that estimated and physiological CS coincide. Nevertheless, when using the estimated CS to prescribe exercise intensity, a careful choice of percent of PS is important to make sure the estimated CS is a very good approximation of the physiological CS.

## Conclusion

Systematic biases were observed between each pair of fitting procedures for the estimations of both CS and *d*′, though negligible when comparing statistically appropriate fitting procedures. The observed differences suggest that a statistically appropriate fitting procedure should be chosen beforehand by the researcher. Indeed, even if these differences could be negligible when prescribing a training session based on exercise intensity, they might vary depending on the data set or the underlying model. This statement is also particularly important for coaches using CS and *d*′ for prescribing training session intensity: the fitting procedure should be maintained over the running seasons. Moreover, we suggest coaches to physiologically verify that the estimated CS represents a very good approximation of the actual CS, to acknowledge the error in the model, and make adjustments when they seem necessary. In addition, this study provides a methodology to determine the statistically appropriate fitting procedures that can be considered based on a specific data set.

## Data Availability

The datasets supporting this article are available on request to the corresponding author.

## Abbreviations

CP: Critical power
CS: Critical speed
*d*′: Distance that can be run above critical speed
LS: Least squares
${s}_{\dot{\mathrm{V}}{\mathrm{O}}_{2}\mathrm{max}}$: Speed associated with maximum oxygen consumption
PS: Peak speed of the incremental test
WLS: Weighted least squares

## Code availability

The code supporting this article is available on request to the corresponding author.
